# Preschool Children with Hearing Loss: Social Communication and Parenting Stress

**DOI:** 10.3390/jpm14010047

**Published:** 2023-12-29

**Authors:** Magdalena Dall, Christoph Weber, Daniel Holzinger, Doris Binder, Johannes Hofer, Sonja Horvarth, Daiva Müllegger, Christoph Rosenthaler, Ruth Zöhrer, Johannes Fellinger

**Affiliations:** 1 Research Institute for Developmental Medicine, Johannes Kepler University Linz, 4020 Linz, Austria; christoph.weber@ph-ooe.at (C.W.); daniel.holzinger@bblinz.at (D.H.); johannes.hofer@bblinz.at (J.H.); daiva.muellegger@bblinz.at (D.M.); johannes.fellinger@bblinz.at (J.F.); 2 Department for Inclusive Education, University of Education Upper Austria, 4020 Linz, Austria; 3 Institute of Neurology of Senses and Language, Hospital of St. John of God, 4020 Linz, Austria; doris.binder@bblinz.at (D.B.); sonja.horvarth@bblinz.at (S.H.); christoph.rosenthaler@bblinz.at (C.R.); 4 Institute of Linguistics, University of Graz, 8010 Graz, Austria; 5 Michael Ogon Laboratory for Orthopaedic Research, Orthopaedic Hospital Speising, 1130 Vienna, Austria; ruth.zoehrer@gmx.at; 6 Division of Social Psychiatry, University Clinic for Psychiatry and Psychotherapy, Medical University of Vienna, 1090 Vienna, Austria

**Keywords:** child, deaf and hard of hearing, parenting stress, problem behavior, social communication, parent–child interaction

## Abstract

Studies on parenting stress (PS) in parents of children with hearing loss (HL) have found relationships between child behavior, language skills and parenting stress. The role of early social communication skills has not been researched before. The aim of this cross-sectional study was to investigate the relationship between child behavior, social communication and PS. The study was performed in a subgroup of a total population sample from the AChild (Austrian Children with Hearing Impairment–Longitudinal Databank) study. Preschool children (n = 81) with all degrees of HL and average cognitive functioning and their families were included, and the Parenting Stress Index (PSI) was used. Through factor component analysis, compound scores for externalizing/internalizing problem behavior and hyperactivity were analyzed. Although mean PS was not elevated, the proportion of those with elevated scores was higher compared with the norm population. There was a strong correlation between child behavior problems and PS (strongest correlation: externalizing problem behavior r = 0.643; *p* < 0.001). All three problem behaviors accounted for 49.7% of the variance in PS. An indirect effect of social communication on PS was almost completely mediated by problem behavior (especially hyperactivity). The importance of social communication development with respect to problem behavior and PS is highlighted.

## 1. Introduction

Building on the stress concepts of Selye [1] and Lazarus [2], Abidin described, in his parenting stress model that includes a child domain (i.e., a combination of child characteristics and behavior) and a parent domain (i.e., parent characteristics and context), that parenting stress arises when there is a perceived mismatch between parental demands and available resources. Dimensions within the child domain challenge parents in specific ways, while dimensions in the parent domain decrease the parental resources needed to successfully manage parenting functions [3].

Parenting stress (PS) in parents of children with hearing loss (HL) has been explored since the early 1990s [4], when Newborn Hearing Screening and early enrolment in early intervention were not implemented universally. The literature on stress in parents of children with HL shows inconsistent results when comparing stress levels with those of parents of typically hearing children. Most of the more recent studies have found that stress levels in parents of children with HL do not differ from those of parents of children with typical hearing [5,6,7,8,9]. These studies all used assessments such as the Parenting Stress Index (PSI) to measure general parenting stress. Two studies [10,11] that found more stress in parents of children with HL used the Family Stress Scale (FSS), which is a context-specific questionnaire for parents of children with HL. This questionnaire specifically includes aspects of communication and educational placement. Quittner et al. 2010 [10] investigated both general and context-specific parenting stress and showed that deafness-specific parenting stress was elevated, while general parenting stress did not differ significantly from that of parents of typically hearing children. Their study mentioned the importance of considering the unique challenges that parents of children with HL have to face, including communication difficulties, educational concerns and maintaining hearing aid devices (HA) [10]. A more recent, retrospective study that included 82 children with HL (more than half of them younger than 36 months of age), found a correlation between maternal stress and additional disabilities, such as a cognitive delay or difficulties in motor skills, as well as maternal educational level [12].

Almost all of the studies investigating factors associated with PS have a cross-sectional design, making it impossible to produce claims about the directionality of the influence. In the field of HL, both directions, the influence of PS on child development and reverse, have been investigated. Behavioral problems and delayed language development are two factors that have been addressed in most studies of children with HL. In a recent systematic review on parenting stress and coping mechanisms in parents of children with HL, the factors related to parenting stress were divided into three categories: parents/family-related, child-related and professionals/service-related. Most factors were grouped into the first two categories [13].

### 1.1. Behavioral Problems

In a systematic review and meta-analysis investigating the association between child behavior problems and PS in clinical groups (autism spectrum disorder, developmental delay, chronic illness), externalizing problem behavior had a stronger influence than internalizing problem behavior on parenting stress [14]. A systematic review that included studies investigating behavior problems in children with HL (wide age range between the studies—children between 2 and 21 years old) found up to one Standard Deviation (SD) higher rates of problem behaviors compared with typically hearing children [15], whereas a more recent study of a large population-based sample of children with HL (9 years of age) showed mean problem behavior ratings within the normal range. A closer look, however, reveals that around twice as many children with HL scored two SDs above the mean of the normative sample [16]. In a study on very young children with HL (18–24 months), no significant difference in maternal stress was found compared with the typically hearing control group. The toddlers with HL showed increased internalizing and withdrawn behavior, and parenting stress was independently associated with internalizing and externalizing problem behavior. There was no interaction effect between parenting stress and HL [6]. In toddlers aged between two and three years, parenting stress was found to be a significant predictor of behavioral problems (externalizing, internalizing and dysregulation), with no significant association between HL and behavioral problems [7]. A study of children with unilateral or mild bilateral HL came to similar results: children with HL on average did not show more behavioral problems, and their parents did not have more parenting stress. Higher levels of parenting stress, however, were related to more child behavior problems [8]. In their study of toddlers with moderate HL, Dirks et al. found no differences in (i) parental stress between parents of children with HL and those of their typically hearing peers and (ii) problem behavior between the two groups of children. An association between more parental stress and more internalizing problem behavior was found in the group of children with HL. There was no significant association between parental stress and externalizing or dysregulating behavior [9].

### 1.2. Delay in Language and Communication Development

As with the question of the direction of influence between behavior and PS (which cannot be answered due cross-sectional design of most studies), it is difficult to make any assumptions on whether high PS leads to difficulties in language development or vice versa. In a large study with 181 toddlers with cochlear implants (CI), higher rates of HL-specific as compared with general PS were shown. A mediation model showed an indirect path from hearing status to PS via language delay and behavioral problems. Expressive language skills accounted for 30% of PS [10]. Blank et al. 2020 [5] investigated the relationship between PS, inhibitory control and language skills and found no significant difference in PS between parents of children with HL and parents of children with typical hearing. However, only for children with HL, there was a negative correlation between receptive language skills and PS. Furthermore, there was a significant correlation between language skills and inhibitory control which was not observed in typically hearing children. More PS was associated with poorer receptive language skills, which were consequently associated with poorer inhibitory control skills. PS explained 15% of the variability in language development [5]. In a sample of 70 school-aged children with CI, again, the overall PS was not significantly different from the normative sample, but parents of children with delayed language development showed significantly higher stress levels [17].

Early social interaction influences both language as well as executive function development. Many studies have looked at language to support self-regulation during executive function tasks in older children, however, in infancy, social interaction already facilitates the development of executive functioning, particularly inhibition and attention control [18]. In longitudinal studies, a relationship between language abilities at the age of 2 years and later executive functioning could be established [19,20]. One study in older children at the ages of 6–11 years who are deaf or hard of hearing (DHH) found that language scores mediated executive function skills but not the other way around [21], while a large, population-based longitudinal study with children between 6–11 years of age found that lower executive function skills were related to higher conduct problems around one year later, which was mediated by theory of mind skills [22].

A recent systematic review showed the link between social communication and mental health, indicating that children and adolescents with social communication difficulties also have higher rates of externalizing and internalizing problem behavior. Additionally, longitudinal studies reported that children with more severe social communication problems have an earlier onset and more persistent course of mental health problems [23]. To date, only a few studies have investigated social communication/pragmatics, and its/their influence on psycho-social behavior in school-aged children and adolescents with HL [16,24,25,26]. In a study of school-aged children fitted with hearing aids with moderate to severe HL, a regression analysis with both structural language and social communication and quality of life as the outcome variable showed only social communication skills to be a significant predictor [25]. A further study with a population-based sample (Longitudinal Outcomes of Children with Hearing Impairment–LOCHI) found that better pragmatic skills were associated with fewer total difficulties and lower hyperactivity scores [16]. The most recent study in school-aged children with HL showed that social communication problems rather than structural language difficulties were associated with emotional problems [26]. Previous studies focused on children and adolescents aged 9 years and above; to our knowledge, early associations between social communication and behavior and mental health in preschool children with HL have not been investigated so far.

### 1.3. Rationale of the Current Study

Previous studies on PS in parents of children with HL have come to mixed results and have focused on either a group of children with a specific degree of HL (unilateral or mild bilateral [8] or moderate HL [9]) or children with CI’s [11,17]. Our cross-sectional study involved a population-based sample of preschool children with permanent HL of various degrees, all of them equipped with HA’s or CI’s, including some with additional disabilities or who were raised bilingually. To the best of our knowledge, this is the first study to investigate early social communication skills as a variable that influences PS in parents of children with HL. The majority of studies that included social communication assessments have been conducted with school-aged children with HL. We, however, assume that the assessment of social communication skills at an earlier age is crucial to fostering social communication during intervention. Within this study, we chose to investigate the effect of child development on PS due to previous evidence from studies in the general population. We do, however, assume reciprocity, although it can neither be confirmed nor rejected by this study.

The following research questions and hypotheses were addressed:

Research question 1

Is there an association between child variables (degree of HLs, use of HA’s or CI’s, IQ, behavioral problems, social communication skills), family variables (number of children, language spoken at home) and PS?

**Hypothesis** **1a.** *PS is associated with child problem behavior and social communication difficulties.*

**Hypothesis** **1b.** *The degree of HL, nonverbal intelligence and the hearing technology used are not associated with PS.*

Research question 2

Considering problem behaviors (internalizing, externalizing, hyperactivity) simultaneously, which type of problem behavior is most strongly associated with PS?

**Hypothesis** **2a.** *Based on recent publications, externalizing problem behavior has the strongest influence on PS.*

**Hypothesis** **2b.** *The degree of HL moderates the associations between problem behavior and PS—with stronger associations between problem behavior and PS with higher degrees of hearing impairment.*

Research Question 3/Hypothesis 3: The association between social communication and PS is mediated by problem behavior.

## 2. Materials and Methods

The AChild (Austrian Children with Hearing Impairment–Longitudinal Databank) study is a longitudinal epidemiological study in Upper and Lower Austria that includes all preschool children (between birth and 66 months) with bi- or unilateral permanent hearing loss of ≥25 dB as the mean HL in the frequencies 0.5, 1, 2 and 4 kHz. The study started in January 2020 and is ongoing. In total, there are six study time points at 9, 18, 27, 36, 48 and 66 months of age. At the start of the study, all children who fulfilled the inclusion criteria could join; therefore, it was possible for children to enter the study at any of the study time points (not only with 9 months of age). Further details of the study can be found in a previously published methods paper [27]. The current study used a subsample of the AChild study, more precisely, all children who had their clinical assessment around the age of 36 or 66 months when PS was assessed by use of the same questionnaire. Families who did not fill out the PS questionnaire were not included in this study. At the time of data analysis, there were a total of 221 children enrolled in the study. The subgroup of children at the time points 36 and 66 months was 106 children.

### 2.1. Assessments

#### 2.1.1. Parenting Stress Index—Short Form (PSI-SF)

The PSI-SF is a widely used self-reported questionnaire that includes 48 items that are divided into 12 subscales and assesses both a child and a parent domain. The German version of the PSI-SF was normed with a group of German mothers of preschool children. The norms are independent of the age of the child. The questionnaire shows good validity and reliability (internal consistency with a Cronbach’s alpha ranging between 0.91 and 0.93 and a test–retest reliability between 0.85 and 0.87) [28]. In this study, the parent domain, comprising seven subscales (depression, attachment, role restriction, competence, social isolation, spouse/parenting partner relationship and health), was used as the outcome variable. The child domain was not used as an outcome variable, since multiple questions address externalizing problem behavior as well as inattention and hyperactivity in similar ways as other measures addressing child behavior. However, the distractibility/hyperactivity subscale was used as an indicator of hyperactivity/inattention. The total score is reported as a T-score, while the subscales are reflected in stanine scores.

#### 2.1.2. Strength and Difficulties Questionnaire (SDQ)

In order to assess the psycho-social wellbeing and behavior of the children included in the study, the parents were asked to complete the SDQ 2–4 (for the children aged 36 months) and the SDQ 4–16 (for the children aged 66 months). This standardized screening questionnaire is commonly used to measure behavior in children. The screening includes five subscales: conduct problems, emotional problems, hyperactivity/inattention, peer-relationship problems and prosocial behavior. Both screening assessments include 25 questions, which are answered on a 3-point Likert scale. In addition to a raw score, two groups are formed: borderline and abnormal. The cut-off scores were chosen such that the norming sample had 10% in the borderline group and 10% in the abnormal group [29]. In this study, three subscales (conduct problems, emotional problems and hyperactivity/inattention) were used as variables.

#### 2.1.3. Child Behavior Checklist 1½-5 (CBCL)

As a second instrument to assess child behavior, the CBCL was used for children aged 66 months. This standardized parent-reported instrument has German norms for the ages 2;0–5;11 years. The questionnaire consists of 100 items that are answered on a three-point Likert scale. There are eight subscales and three combined scales (externalizing, internalizing and total), which give the t-score results. The assessment shows good internal consistency, with Cronbach’s alphas between 0.82 and 0.92 [30]. The internalizing domain and the aggressive behavior and attention problems subscales were used in this study.

#### 2.1.4. Behavior Rating Inventory of Executive Functioning—Preschool Version (BRIEF-P)

The German version of the BRIEF-P, “Verhaltensinventar zur Beurteilung exekutiver Funktionen für das Kindergartenalter”, was used for all children. BRIEF-P is a standardized questionnaire that measures various aspects of executive functioning in children between the ages of 2;0 and 6;0 years in the form of parent or educator reports. The BRIEF-P is composed of 63 items that are answered using a three-point Likert scale: five clinical scales, three broad indexes and one composite score or Global Executive Composite, which provides the t-score results. For the purpose of the AChild study, only the parent version was used. The questionnaire shows good internal consistency for the scales of the parent version, with Cronbach’s alphas between 0.75 and 0.59 [31].

#### 2.1.5. Language Use Inventory (LUI)

To assess social communication skills in the younger children at 36 months, the LUI was used, which is a standardized parent-reported questionnaire originally published in English for children between the ages of 18 and 47 months. For the purpose of the AChild study, the questionnaire was translated into German and backtranslated. Currently, the original norms based on Canadian children are still used. The German version includes 181 items grouped into 14 subscales, as well as a total score. The questionnaire shows good internal consistency, with Cronbach’s alphas ranging between 0.53 and 0.99 [32].

#### 2.1.6. Children’s Communication Checklist-2 (CCC-2)

For the older children, at 66 months, social communication skills were assessed using the CCC-2. This standardized questionnaire is parent-reported and includes 70 questions, which are divided into 10 subscales [33]. Since the questionnaire has not yet been normed in a German-speaking sample, the US norms were used for this study. Even though both the LUI and CCC-2 assess social communication skills reported by parents, there are differences in the communicative functions and nonverbal behaviors measured (also related to the different age groups) and in items referring to social cognition or even autism-specific behaviors.

#### 2.1.7. Snijders-Oomen NonVerbal Intelligence Test 2½-7 (SON-R)

To assess cognition, the SON-R was applied by a psychologist in a clinical setting. The SON-R is a standardized assessment that has German norms and consists of six subtests, which are combined into a total IQ score. The mean internal consistency of the subtest is 0.70 and the total IQ is 0.90 [34].

### 2.2. Audiological Measures

The degree of HL at diagnosis was either assessed through frequency-specific auditory brainstem response (BERA) or auditory steady-state response (ASSR). From this, the Fletcher Index is reported as the average at 0.5, 1, 2 and 3 kHz in the better ear. This is then subsequently categorized in line with the classification of degree of HL from the WHO: mild < 31 dB, moderate 31–60 dB, severe 61–80 dB and profound > 81 dB [35]. Additionally, information on the use of HA’s or CI’s is given.

### 2.3. Statistical Analysis

In this study, there are several scales available that measure the same constructs (e.g., SDQ and CBCL). To reduce the number of variables and, thus simplify the analyses, we applied a two-step approach.

First, we used confirmatory factor analysis (CFA) to estimate factor scores based on all available indicators per construct for hyperactivity/inattention, externalizing and internalizing problems. We evaluate the fit of the CFA models and the reliability of the factors. Moreover, to evaluate the quality of the factor score, we estimated the factor score determinacy index (FDI, i.e., the correlation of the factor score with the latent variable), which should be >0.80 for research purposes [36]. Notably, given conceptually different measures of social communication, we refrained from estimating a factor score for social communication but retained the CCC-2 and the LUI as separate measures.

Second, we used the factor scores in subsequent analyses to test the hypotheses of this study. To answer research question 1, we used correlation analysis. In regard to hypothesis 2a, we regressed the PS index (parent domain) on the factor scores for hyperactivity/inattention, externalizing and internalizing problems. Given the expected high correlations among the predictors, standardized regression coefficients may be flawed estimates of predictor importance [37]. Thus, we also applied relative weight analysis (RWA) [37], which has been proposed as a useful supplement to regression analysis. RWA properly partitions the explained variance among correlated predictors and thus allows us to better understand the role of the three problem behaviors in predicting parental stress. Regarding hypothesis 2b, we used moderated regression analysis [38]. We ran separate models for the three problem behavior measures and the two social communication measures as predictors and entered the Fletcher index and the interaction term predictor × Fletcher index as further independent variables. A significant effect of the interaction term would indicate that the degree of hearing loss moderates the associations between a predictor and parental stress. Finally, to test research question 3, we applied mediation analysis using a structural equation modeling (SEM) framework [39]. In detail, we tested whether there is an indirect effect of social communication on PS that is mediated by problem behavior. To assess the significance of the indirect effect, we used bias-corrected bootstrapping with 10.000 bootstrap samples. An indirect effect is regarded as statistically significant if the 95% confidence interval does not include 0. We used the following guidelines to evaluate model fit in CFA and SEM [40]: χ^2^/df ≤ 2 indicates a good and χ^2^/df ≤ 3 an acceptable fit, and a Comparative Fit Index (CFI) ≥ 0.975 suggests a good fit and CFI ≥ 0.95 an acceptable fit. Standardized Root Mean Residual (SRMR) values ≤ 0.05 are indicative of a good fit, and SRMR ≤ 0.10 is regarded as an acceptable fit. Finally, a Root Mean Square Error of Approximation (RMSEA) ≤ 0.05 indicates a good and an RMSEA ≤ 0.08 an acceptable fit.

Descriptive analyses were conducted using SPSS 28. The relative weight analysis was conducted using the Relative Importance Shiny App [41]. All other analyses were conducted using Mplus 8 [42].

## 3. Results

### 3.1. Participants

In total, 81 children were included, 40 of whom were assessed at the age of 36 months (+/−3 months) and 41 at 66 months (+/−6 months). There were slightly more boys (59.3%) in the sample. The degree of HL was heterogeneous, with 15.2% of the children having mild, 43.0% moderate, 6.3% severe or 10.0% profound HL. A total of 26.2% of the children had unilateral and 73.8% bilateral HL. Similar to the total AChild sample, 76.5% used HA’s, while 22.2% used CI’s. One child used both, CI, and on the contralateral side, an HA. Almost all children were diagnosed at an early age, with a mean of 12.0 months (SD = 14.5) and were enrolled in early intervention at a mean age of 18.3 months (SD = 1.8). With 23.5% of children growing up bilingually, our sample differed significantly from the sample that should be available for the used age groups with about one-third bilingual children (χ^2^(1) = 11.0, *p* < 0.001; Phi = 0.340). Thus, the parents of bilingual children were more likely to not fill in the PSI. The children had an average level of intellectual functioning (mean = 99.9; SD = 15.2). There was no significant difference in cognition from the sample that should have been available. Table 1 gives a detailed description by age.

### 3.2. Confirmatory Factor Analysis and Factor Score Estimation

For hyperactivity/inattention, four indicators are available for factor score estimation: the SDQ hyperactivity subscale, the CBCL hyperactivity/inattention subscale, the PSI distractibility/hyperactivity subscale, and the BRIEF-P total score. The CFA showed an acceptable-to-good fit, as indicated by an χ^2^(2) = 4.16, *p* = 0.13, CFI = 0.982 and an SRMR = 0.036. Only the Root Mean Square Error of Approximation (RMSEA) of 0.115 indicated a poor fit of the model to the data. However, RMSEA has been shown to over-reject models with small df, especially when the sample size is small, and thus, it is recommended to not even report RMSEA in such cases [43]. Thus, we subsequently do not report RMSEA. The hyperactivity/inattention factor shows acceptable reliability (MacDonald’s ω = 0.779). FDIs for the hyperactivity/inattention factor score range from 0.82 to 0.95 (note, there is an FDI range due to missing data of the indicators. Factor scores are computed as long as there is valid information on at least one indicator. The FDI range refers to different missing data patterns). For externalizing and internalizing problems, there are only two indicators available per construct (SDQ conduct problems and CBCL aggressive behavior for externalizing problems and SDQ emotional problems and CBCL internalizing problems for internalizing problems). A CFA with only two indicators is not identified, and thus, model parameters and factor scores cannot be computed. To deal with this problem, we constrained the two factor loadings to be equal, which results in a saturated model (i.e., df = 0) that allows model and factor score estimation. However, saturated models yield a “perfect fit”; thus, meaningful testing of the model fit is not possible. Reliability (MacDonald’s ω) is good for externalizing problems (ω = 0.877) and acceptable for internalizing problems (ω = 0.773). FDIs are largely in an acceptable range (0.82 to 0.95 for externalizing and 0.79 to 0.88 for internalizing problems). For hyperactivity/inattention, externalizing and internalizing problems factor scores (in a z-metric, i.e., M = 0, SD = 1) were saved for use in subsequent analyses.

### 3.3. Descriptive Statistics for the Main Study Variables

Table 2 shows the descriptive statistics for the main study variables. The proportion of children with elevated and abnormal externalizing problem behavior assessed by the SDQ was about one-third higher than in the normative population (32.5% vs. 20%). However, in our sample, the proportion of children with elevated rates of internalizing problem behavior was somewhat smaller than in the normative sample (8.2% vs. 20%). The percentage of children exhibiting elevated and abnormal rates of hyperactivity measured with the SDQ was slightly lower than in the norming population (14.9% vs. 20%). In terms of social communication skills, it is evident that the children were still lagging behind their hearing peers, with a mean percentile score of about 18–19 on the LUI and CCC-2. The mean score for the parent domain of the PSI was comparable to that of the general population, but there were more parents with elevated and highly elevated PS scores.

### 3.4. Correlations with PS

The results regarding research question 1 are shown in Table 3. First, PS is strongly correlated with child problem behavior—with the highest correlations between PS and externalizing problems (r = 0.643, *p* < 0.001), followed by hyperactivity (r = 0.606, *p* < 0.001) and internalizing problems (r = 0.582, *p* < 0.001). Second, there is a significant correlation of moderate size between PS and social communication (rLui = −0.443, *p* < 0.01). Third, the degree of hearing loss (Fletcher index) is not associated with the level of PS (r = 0.021, *p* > 0.05). Finally, all other child variables (age, gender, age of diagnosis) are not associated with PS.

### 3.5. Partial Effects of Problem Behavior on PS

The regression analysis indicates that, taken together, the three problem behaviors account for 49.7% of the variance in PS (Table 4). The regression coefficients are all significant at *p* < 0.05. The standardized coefficient indicates that externalizing problems have the strongest effect (β = 0.295) on PS, followed by internalizing problems (β = 0.267) and hyperactivity (β = 0.250). The RWA confirms that externalizing problems explain the largest fraction of variance in PS (raw weight = 0.179; 95% confidence interval [0.097, 0.281]), accounting for 36.1% (relative weight) of the total variance explained, and indicates that hyperactivity (raw weight = 0.161; 95% confidence interval [0.062, 0.267]; relative weight = 32.4%) accounts for a larger share of variance than internalizing problems (raw weight = 0.157; 95% confidence interval [0.058, 0.272]; relative weight = 31.5%). However, as indicated by confidence intervals including 0, the differences between the raw weights are not significant (externalizing problems vs. internalizing problems 95% confidence interval [−0.182, 0.130]; externalizing problems vs. hyperactivity 95% confidence interval [−0.154, 0.100], internalizing problems vs. hyperactivity 95% confidence interval [−0.182, 0.151]). In summary, the three problem behavior dimensions are of similar importance in predicting PS.

### 3.6. Degree of Hearing Loss as Moderator of Problem Behavior Effects

The moderation analyses indicate that the degree of hearing loss does not moderate the associations between PS and (a) problem behaviors and (b) social communication, respectively (see Table 5). However, in line with the correlation analyses, all the main effects of problem behavior and social communication are significant, and the degree of hearing loss is not associated with PS. None of the interaction effects reached statistical significance.

### 3.7. Problem Behavior as Mediator of Social Communication Effects

**Figure 1:**
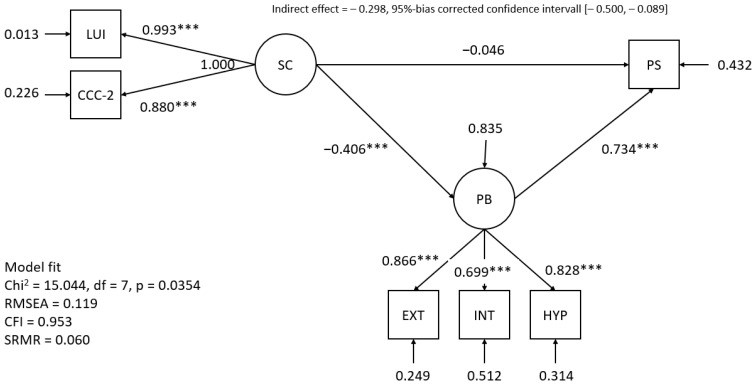
Results of the mediation analysis (standardized estimates). Notes. SC = social communication, PS = parenting stress, EXT = externalizing problems, INT = internalizing problems, HYP = hyperactivity, PB = problem behavior. *** *p* < 0.001.

In order to determine whether problem behavior mediates the effect of social communication on PS, we tested a latent mediation model with LUI and CCC-2 being indicators of a latent variable social communication and externalizing problems/internalizing problems and hyperactivity being indicators of a latent problem behavior factor (see Figure 1). Overall, the model shows an acceptable fit, as indicated by a Chi^2^/df = 2.15, CFI = 0.953 and SRMR = 0.060. The results show a significant effect of social communication on problem behavior (stand. b = −0.406, *p* < 0.001). Thus, lower social communication skills are associated with higher levels of problem behavior. Additionally, in line with the results above, there is a strong effect of problem behavior on PS (stand. b = 0.734, *p* < 0.001). The direct effect of social communication on PS is near 0 and not significant (stand. b = −0.046, *p* > 0.05). However, the indirect effect of social communication on PS is of moderate size (stand. b = −0.298) and—as indicated by the bias-corrected bootstrapped confidence interval not including 0 (95% confidence interval [−0.500, −0.089])—also statistically significant. Thus, the effect of social communication is almost completely mediated by problem behavior.

Supplementary analyses that did not consider a total problem behavior variable but used the three problem scales separately as mediators showed that hyperactivity plays the most important role in mediating the effects of social communication on PS. Considering hyperactivity as a mediator, no significant indirect effect and no direct effect were found. Thus, the direct effect of social communication is largely mediated by hyperactivity. For externalizing problems, there is a significant indirect effect (b = −0.145, bc Bootstrap 95% confidence interval [−0.299, −0.012]) but also a marginally significant direct effect (b = −0.206, *p* < 0.10). For internalizing problems, both the direct and the indirect effects are only marginally significant. For details see Table 6.

## 4. Discussion and Conclusions

This cross-sectional study investigated child variables, including externalizing and internalizing behavioral problems and social communication difficulties, and their influences on PS in a population-based sample of preschool children with HL.

Overall, there was no significant difference in the mean of perceived PS between our sample and the normative data for parents of typically hearing children. However, there was a higher proportion of parents scoring in the highly elevated range (7.4%).

Our sample showed higher rates of behavioral problems than the normative sample. The largest difference was found in the externalizing problem behavior rate—with a mean of 20.3% of children scoring in the abnormal range, and in the older group, even 24.3% were in the abnormal category, compared with 10% in the total population. The internalizing problem behavior was slightly lower compared with the normative sample (6.8% vs. 10%). Also, in this group, the older children showed slightly higher rates (8.1%) compared with the younger children. The hyperactivity/inattention rate for the total sample showed higher rates in the abnormal category (12.2% vs. 10%). When looking at the older children, it becomes evident that the rates are rising, with 18.9% in the abnormal group. Higher rates of attention difficulties in children with HL are not unusual [44,45], but our sample was much younger than those of other studies, and this is the first time this has been described in preschoolers. While other studies in children with HL have reported behavior and language scores that did not differ significantly from those of hearing children [9,16], our sample showed elevated rates of behavioral problems and social communication difficulties. Notably, the other study was conducted on nine-year-old school children, while our study focused on preschool children. No other study has hitherto investigated social communication skills in such young children with HL. The results for social communication skills (below the 20th percentile) show that the children in this sample are lagging behind typically hearing peers. These difficulties in social communication existed even though the children in our sample were all diagnosed and enrolled early in family-centered early intervention. They presented with difficulties concerning the functional use of language, which should be recognized and worked on.

Around 50% of PS could be explained by the three problem behavior variables, each of which contributed around one-third to the explained variance. This result confirms hypothesis 1abut contradicts hypothesis 2a expecting stronger influences of externalizing problem behaviors on PS. IQ and degree of hearing loss did not correlate with PS.

The path model presented in this study showed an indirect effect of social communication skills on PS via problem behavior but no indirect effect of the degree of HL. This finding contradicts hypothesis 2b but confirms our last hypothesis and highlights the importance of social communication rather than the degree of HL on child development in children with HL. Especially, the effect of early social communication skills on hyperactivity in these children should be considered when planning early intervention models for children with HL. Cross-sectional studies in typically hearing children with ADHD have found significant correlations between pragmatic language difficulties and ADHD symptoms [46,47]. Higher rates of pragmatic difficulties in children with ADHD compared with typically developing children were also shown in a recent systematic review including 34 studies [48].

Our path model is similar to the one of Quittner et al. [10], who found a link between language delays and PS via child behavior problems. In addition to the importance of social communication skills (besides language skills) for child mental health, as found by previous studies, our findings also highlight the key role played by the functional use of language for both child behavior and PS. As proposed by Morgan et al., 2021 [18], early social interaction, such as joint attention or turn-taking, has an influence on the early development of executive functions. Particularly in children with HL who have hearing parents, it is important for their parents to respond sensitively and consistently to their children’s attempts for early communication. Studies showed that in parents with HL, there is higher intuitive synchronicity in their interaction with their child with HL, and the children develop typical intersubjectivity [49], which further on is linked to an increase in executive function skills [50].

Our study has several clinical implications. First, assessment of social communication skills and behavior in children with HL is key. Even though some studies did not report elevated levels of problem behavior [9], we found a significantly higher proportion of children within the clinically relevant group in externalizing problem behavior and hyperactivity of the SDQ. Consequently, early intervention programs should focus on behavior and emotion regulation. This study shows that social communication deficits are not only associated with child behavior problems but also with higher PS, which can, in turn, have negative effects on parent–child interaction. Therefore, incorporating social communication intervention is crucial as a strategy to enhance self-regulation.

This study has some limitations. Firstly, due to its cross-sectional design, no claims can be made regarding the direction of the influence of PS and child variables, such as behavioral problems and social communication. The assumed direction was based on previous publications. We are, however, aware that undoubtedly there are bidirectional influences. As the longitudinal AChild data set is continuously being expanded, we will be able to identify early predictors of child behavior and PS that can be targeted by early identification and intervention. Secondly, the current study is based on a given sample size that was available at the time the preparation of the manuscript started. Therefore, the sample size was limited, which also entails power issues. In order to evaluate the power of the current study post hoc and to provide sample size guidelines for studies aiming to replicate our findings, we conducted a Monte Carlo simulation study (10.000 replications) for the most complex analysis of this paper, i.e., the mediation model shown in Figure 1 [51]. However, using the reported sample estimates as effect size measures for the power analysis is a flawed and repeatedly criticized approach that yields biased power estimates [52,53]. Therefore, we used values one standard error below the sample estimate (i.e., the 16% percentile of the estimate) as effect size measures [53]. The power for the effect of behavioral problems on PS is high at 1.00. However, the power for the effect of social communication on problem behavior (0.493) and the indirect effect (0.462) is below the desired value of 0.80. To assure sufficient power in future studies, a sample size of n = 180 is required, which results in power estimates > 0.80 for all effects that were significant in the current study. Thirdly, even though the sample was taken from a population-based study, there were fewer bilingual children included than in the total study and the total population. The reasons for this were discussed, and more attention should be paid to this group of children during ongoing data collection. Lastly, since some assessments used have not yet been normed in a German-speaking sample, British or American norms were used.

Future studies should investigate PS within a longitudinal research design in order to obtain more concrete answers to the question of the directionality of influence. Another interesting aspect is the inclusion of parental self-efficacy—possibly related to the quality of parental interaction with the child and associated social communication skills—as a resource that can prevent PS. This should also be investigated over time to assess the possibility of it being a factor that can be influenced by family-centered early intervention.

Children with HL on average lag behind their typically hearing peers in social communication. Due to associations between social communication skills and reduced behavior problems, and consequently PS, this study points to the importance of monitoring and enhancing social communication skills in children with HL from very early on.

## Figures and Tables

**Table 1:** Participant and Family Characteristics.

	36 Months	66 Months	Total	Min–Max ^b^
Number of children	40	41	81	
Age ^a^ (months)—m (SD)	38.13 (2.2) *	66.3 (5.7) *	52.4 (14.8) *	35–73
Sex, male—n (%)	23 (57.5)	25 (61.0)	48 (59.3)	
**Intellectual functioning, mean (SD)**	103.4 (15.3) *	96.4 (14.4) *	99.9 (15.2)	56–135
Bayley	103.6 (20.7)	97.4 (14.5)	101.5 (18.9)	65–145
SON	101.5 (14.5)	96.7 (14.9)	98.9 (14.8)	55–131
**Number of siblings—n (%)**				
0	17 (42.5)	18 (45.0)	35 (43.8)	
1	14(35.0)	16 (40.0)	30 (37.5)	
2	5 (12.5)	5 (12.5)	10 (12.5)	
3 or more	4 (10.0)	1 (2.5)	5 (6.3)	
**Primary language spoken at home—n (%)**				
German	29 (72.5)	33 (80.5)	62 (76.5)	
Bilingual (two or more spoken languages)	11 (27.5)	8 (19.5)	19 (23.5)	
**Degree of HL bilateral (in the better ear)—n (%)**				
Mild	6 (15.4)	6 (15.0)	12 (15.2)	
Moderate	16 (41.0)	18 (45.0)	34 (43.0)	
Severe	0 (0.0)	5 (12.5)	5 (6.3)	
Profound	4 (10.3)	4 (10.0)	8 (10.1)	
**Fletcher Index—mean (SD)**	44.7 (26.5)	53.7 (26.8)	49.2 (26.9)	5–115
**Laterality—n (%)**				
Bilateral	26 (66.7)	33 (80.5)	59 (73.8)	
Unilateral	13 (33.3)	8 (19.5)	21 (26.2)	
**HA supply**				
CI bilateral	7 (17.5)	10 (24.4)	17 (21.0)	
CI unilateral	1 (2.5)	0 (0.0)	1 (1.2)	
CI unilateral and HA unilateral	1 (2.5)	0 (0.0)	1 (1.2)	
HA aid unilateral	11 (27.5)	7 (17.1)	18 (22.2)	
HA bilateral	20 (50.0)	24 (58.5)	44 (54.3)	
Age at diagnosis (months)—mean (SD)	8.7 (10.1) *	16.0 (17.2) *	12.2 (14.3) *	0–73
Age at start of family intervention (months)—mean (SD)	11.6 (11.8) *	25.8 (24.1) *	18.8 (20.2) *	0–64
Age at amplification of hearing aid (months)—mean (SD)	11.7 (11.4) *	26.5 (22.5) *	18.5 (18.7) *	0–63

Note. For 8 children, data were available at both ages, 36 months and 66 months. For these children, we randomly selected one time point to be used in the analyses. * indicates significant (*p* < 0.05) differences between age groups. ^a^ age at EBI completion. ^b^ Minimum and maximum in the total sample for continuous variables. Bold variables are sub headers.

**Table 2:** Descriptive statistics for the main study variables.

	36 Months	66 Months	Total	Min–Max ^a^
**Externalizing problem behavior**				
Externalizing factor score, mean (SD)	0.02 (0.91)	−0.02 (0.89)	0.00 (0.90)	−1.28–3.32
SDQ conduct problems, mean (SD)	2.5 (2.1)	2.2 (1.7)	2.4 (1.9)	0–10
Borderline n (%)	5 (13.5)	4 (10.8)	9 (12.2)	
Abnormal n (%)	6 (16.2)	9 (24.3)	15 (20.3)	
CBCL aggressive behavior, raw score, mean (SD)	-	8.1 (6.8)	8.1 (6.8)	0–29
**Internalizing problem behavior**				
Internalizing factor score, mean (SD)	−0.14 (0.73)	0.14 (0.93)	0.00 (0.84)	−1.12–2.61
SDQ emotional symptoms	1.1 (1.3)	1.7 (1.6)	1.4 (1.5)	0–6
Borderline n (%)	0 (0.0)	1 (2.7)	1 (1.4)	
Abnormal n (%)	2 (5.4)	3 (8.1)	5 (6.8)	
CBCL internalizing problems T-score, mean (SD)	-	49.4 (10.5)	49.4 (10.5)	29–72
**Hyperactivity**				
Hyperactivity factor score, mean (SD)	−0.09 (0.94)	0.09 (0.97)	0.00 (0.95)	−1.50–2.82
SDQ hyperactivity/inattention mean (SD)	3.1 (2.1)	3.9 (2.6)	3.5 (2.4)	0–10
Borderline n (%)	1 (2.7)	1 (2.7)	2 (2.7)	
Abnormal (%)	2 (5.4)	7 (18.9)	9 (12.2)	
CBCL attention problems, raw scores, mean (SD)	-	2.0 (1.9)	2.0 (1.9)	0–6
PSI hyperactivity, mean stanine score (SD)	5.1 (2.0)	5.3 (1.6)	5.2 (1.8)	2–9
BRIEF-P hyperactivity, T-score, mean (SD)	44.8 (11.2)	47.6 (10.9)	46.3 (11.0)	31–85
**Social Communication (pragmatics)**				
LUI total percentile score mean (SD)	17.9 (21.6)			0–70
CCC-2 total percentile score mean (SD)		18.9 (21.8)		0–72
**Parenting stress**				
PSI Parenting stress, mean T-score	51.3 (11.9)	51.6 (10.9)	51.4 (11.3)	30–70
Elevated T-score ≥ 60, n (%)	8 (20.0)	7 (16.9)	15 (18.4)	
Highly elevated T-score ≥ 70, n (%)	4 (10.0)	2 (4.9)	6 (7.4)	

Note. ^a^ Minimum and maximum in the total sample for continuous variables. Bold variables are sub headers.

**Table 3:** Correlations of Study Variables.

		(1)	(2)	(3)	(4)	(5)	(6)	(7)	(8)	(9)	(10)	(11)	(12)	(13)	(14)	(15)	(16)	(17)
Parental stress	(1)	1																
Externalizing problems	(2)	0.643 ***	1															
Internalizing problems	(3)	0.582 ***	0.620 ***	1														
Hyperactivity	(4)	0.606 ***	0.730 ***	0.528 ***	1													
LUI	(5)	−0.443 **	−0.251	−0.333 *	−0.498 ***	1												
CCC-2	(6)	−0.250	−0.282	−0.211	−0.483 **	0.829 *	1											
Age	(7)	−0.007	−0.011	0.173	0.116	−0.189	0.054	1										
Male	(8)	0.130	0.163	0.074	0.172	−0.186	−0.141	0.045	1									
IQ	(9)	−0.063	−0.093	−0.006	−0.295 **	0.470 **	0.482 **	−0.232 *	−0.221	1								
Number siblings	(10)	−0.105	−0.087	−0.098	−0.016	−0.007	−0.258	−0.103	0.087	−0.076	1							
Multilingual	(11)	−0.113	−0.086	−0.052	−0.048	0.001	−0.07	−0.112	−0.193	−0.077	0.149	1						
Bilateral	(12)	0.022	−0.056	0.038	0.039	−0.248	−0.118	0.141	0.093	−0.039	0.065	−0.134	1					
Fletcher index	(13)	0.021	−0.103	−0.008	−0.064	−0.164	−0.089	0.101	−0.017	−0.038	−0.345 **	0.047	0.473 ***	1				
Cochlear implant	(14)	0.019	−0.115	−0.031	−0.006	−0.257	−0.039	−0.047	−0.075	−0.081	−0.259	0.175	0.266	0.658 ***	1			
Hearing aid	(15)	−0.045	0.095	0.003	−0.003	0.311	0.039	0.021	0.101	0.083	0.271	−0.125	−0.253	−0.667 ***	−0.966	1		
Age at amplification HA	(16)	0.003	0.033	0.088	0.062	−0.394	−0.206	0.441	−0.016	−0.16	0.159	−0.033	0.157	−0.143	−0.25	0.25	1	
Age at diagnosis	(17)	0.036	0.074	0.068	0.202	−0.55	−0.471	0.267	−0.014	−0.15	0.163	0.012	0.098	0.109	0.005	−0.03	0.651	1
Age at intervention start	(18)	0.11	0.001	0.125	0.209	−0.601	−0.41	0.387	0.131	−0.317	0.207	0.023	0.231	0.067	−0.128	0.104	0.701	0.723

Note. Ns varies from 8 (r between LUI and CCC-2) to 81. *** *p* < 0.001, ** *p* < 0.01, * *p* < 0.05.

**Table 4:** Regression and Relative Weight Analyses for PS.

	OLS Regression		Relative Weight Analysis			
	B (SE)	Β	Raw Weights			Relative Weights
			Estimate	95% Confidence Interval LL	95% Confidence Interval UL	
Intercept	65.345 (1.758)					
Externalizing problems	7.197 * (3.153)	0.295	0.179	0.097	0.281	36.11%
Internalizing problems	6.932 * (2.706)	0.267	0.157	0.058	0.272	31.54%
Hyperactivity	5.752 * (2.746)	0.250	0.161	0.062	0.267	32.35%
R^2^			0.497			100%

Note. The raw weight estimates sum to the total variance explained (i.e., R^2^). The relative weights indicate the percentage of variance that is explained by a predictor. LL = lower limit, UL = upper limit. Note. * *p* < 0.05.

**Table 5:** Moderation Analysis.

	Predictors				
	Externalizing Problems	Internalizing Problems	Hyperactivity	Social Communication (LUI)	Social Communication (CCC-2)
	B (SE)	B (SE)	B (SE)	B (SE)	B (SE)
Intercept	0.000 (0.111)	0.000 (0.111)	0.000 (0.111)	0.000 (0.111)	0.000 (0.111)
Predictor	0.663 *** (0.077)	0.584 *** (0.101)	0.614 (0.084)	−0.399 ** (0.148)	−0.312 * (0.145)
Fletcher Index (Degree of Hearing Loss)	0.048 (0.082)	0.017 (0.092)	0.077 (0.106)	−0.025 (0.102)	−0.029 (0.116)
Predictor × Fletcher Index	−0.090 (0.092)	−0.037 (0.110)	0.050 (0.132)	0.145 (0.144)	0.010 (0.177)
R^2^	0.424	0.340	0.373	0.209	0.096

Note. *** *p* < 0.001, ** *p*< 0.01, * *p* < 0.05.

**Table 6:** Total, direct and indirect effects of social communication on PS.

	Effects of Social Communication on PS			
	Total Effect	Direct Effect	Indirect Effect	Bc Bootstrap 95% Confidence Interval
Mediators	B (SE)	B (SE)	B (SE)	
Externalizing problems	−0.352 ** (0.124)	−0.207 (*) 0.108	−0.145 * (0.069)	[−0.299, −0.012]
Internalizing problems	−0.352 ** (0.122)	−0.221 (0.123)	−0.132 (*) (0.077)	[−0.301, 0.009]
Hyperactivity	−0.343 (0.121)	−0.042 (0.128)	−0.301 *** (0.078)	[−0.475, −0.166]

Note. *** *p* < 0.001, ** *p*< 0.01, * *p* < 0.05.

## Data Availability

Data presented in this study are not publicly available.
